# Metal Graphitic Nanocapsules for Theranostics in Harsh Conditions

**DOI:** 10.3389/fchem.2022.909110

**Published:** 2022-05-13

**Authors:** Yanxia Yang, Shengkai Li, Hongxiu Bu, Xin Xia, Long Chen, Yiting Xu, Zhuo Chen

**Affiliations:** ^1^ Aptamer Engineering Center of Hunan Province, Molecular Science and Biomedicine Laboratory (MBL), State Key Laboratory of Chemo/Bio–Sensing and Chemometrics, Hunan Provincial Key Laboratory of Biomacromolecular Chemical Biology, College of Chemistry and Chemical Engineering, Hunan University, Changsha, China; ^2^ Faculty of Science and Technology, University of Macau, Macau, China; ^3^ Key Laboratory of Theoretical Organic Chemistry and Function Molecule, Ministry of Education, School of Chemistry and Chemical Engineering, Hunan University of Science and Technology, Xiangtan, China

**Keywords:** metal graphitic nanocapsules, theranostics, harsh condition, gastric environment, stability

## Abstract

Metal nanoparticles (NPs) with superior physicochemical properties and biocompatibility have shown great potential in theranostics. However, metal NPs show poor stability in some harsh conditions such as strong acid, oxidation, corrosion and high-temperature conditions, which limits their extensive bioapplications. To address such issue, a variety of superstable metal graphitic nanocapsules with the metal cores confined in the nanospace of few-layer graphitic shell have been developed for biodetection and therapy in harsh conditions. In this mini-review, we summarize the recent advances in metal graphitic nanocapsules for bioapplications in harsh conditions. Firstly, their theranostic performance in non-intrinsic physiological harsh environment, including oxidation, corrosion and high-temperature conditions, is systematically discussed. Then, we highlight their theranostic performance in the harsh stomach condition that is strong acidic and pepsin-rich. It is expected that this review will offer inspiration to facilitate the exploitation of novel theranostic agents that are stable in harsh conditions.

## Introduction

Metal nanoparticles (NPs) with different compositions and unique physicochemical properties are powerful tools for various biodetection and therapy (Azharuddin et al., 2019; Kim et al., 2019; Ma et al., 2021b). However, metal NPs show unsatisfactory stability in some non-intrinsic physiological harsh conditions (including strong oxidation, corrosion and high-temperature conditions) as well as harsh stomach condition (including strong acid (pH 0.9-1.5) and pepsin-rich conditions), and these harsh conditions hinder their broad applications in biomedicine. Hence, it is of great significance to develop effective strategies to improve the stability of metal NPs for reliable disease diagnosis and treatment in harsh conditions. The encapsulation of metal NPs is one of the most effective strategies to promote their stability (Gao et al., 2021). Currently, inert inorganic coating (such as silica (Li et al., 2010; Żygieło et al., 2021), titanium dioxide (Jin et al., 2021; Perumal et al., 2021) and graphene oxide (Xu et al., 2020; Kasztelan et al., 2021)) protection and organic coating (such as polyvinylpyrrolidone (Mirzaei et al., 2017)and lipids (Hsu et al., 2018)) functionalization strategies have been widely used to prevent the metal NPs from damage under external environments. However, these strategies make it difficult to completely isolate metal NPs from harsh conditions without affecting their inherent properties and functions.

Metal graphitic nanocapsules, a new type of nanomaterials with metal cores confined in the nanospace of few-layer graphitic shell prepared by the chemical vapor deposition method, show superior stability in harsh conditions (Li et al., 2019, 2022; Liu et al., 2020; Tang et al., 2021; Zhu et al., 2021). The graphitic shell acts as an inert layer to protect the unique physicochemical properties and intact functions of the metal core. Moreover, the graphitic shell with a large specific surface area and delocalized *π* electronic structure offers a robust platform for targeted molecules or drugs loading, and it also acts as a stable Raman label or internal standard molecule for reliable Raman bioanalysis. Benefiting from the ultra-high stability of graphitic layer and versatility of metal cores, a variety of theranostic applications in harsh conditions have been implemented by metal graphitic nanocapsules in the past few years. In this mini-review, we first introduce the theranostic advances of the metal graphitic nanocapsules in non-intrinsic physiological harsh environment, including oxidation, corrosion and high-temperature conditions. Then, we highlight the theranostic performance of the metal graphitic nanocapsules in the strong acid (pH 0.9-1.5) and pepsin-rich stomach conditions. Finally, the potential challenge and development direction of their theranostic applications in harsh conditions are further discussed. We expect this review will attract readers to facilitate the exploitation of novel theranostic agents that are stable in harsh conditions.

### Theranostics in Non-intrinsic Physiological Harsh Conditions

Metal NPs-based theranostic agents are commonly subjected to a variety of non-intrinsic physiological harsh conditions, including strong oxidation, corrosion and high temperature conditions that exists during the occurrence, development and theranostics of diseases. These harsh conditions affect the stability of metal NPs to a certain extent, making direct biodetection and therapy challenging. Core-shell structured metal graphitic nanocapsules that integrated the multifunctional metal core in the nanospace of inert graphitic shell demonstrate exceptional theranostic potential in harsh conditions.

**FIGURE 1:**
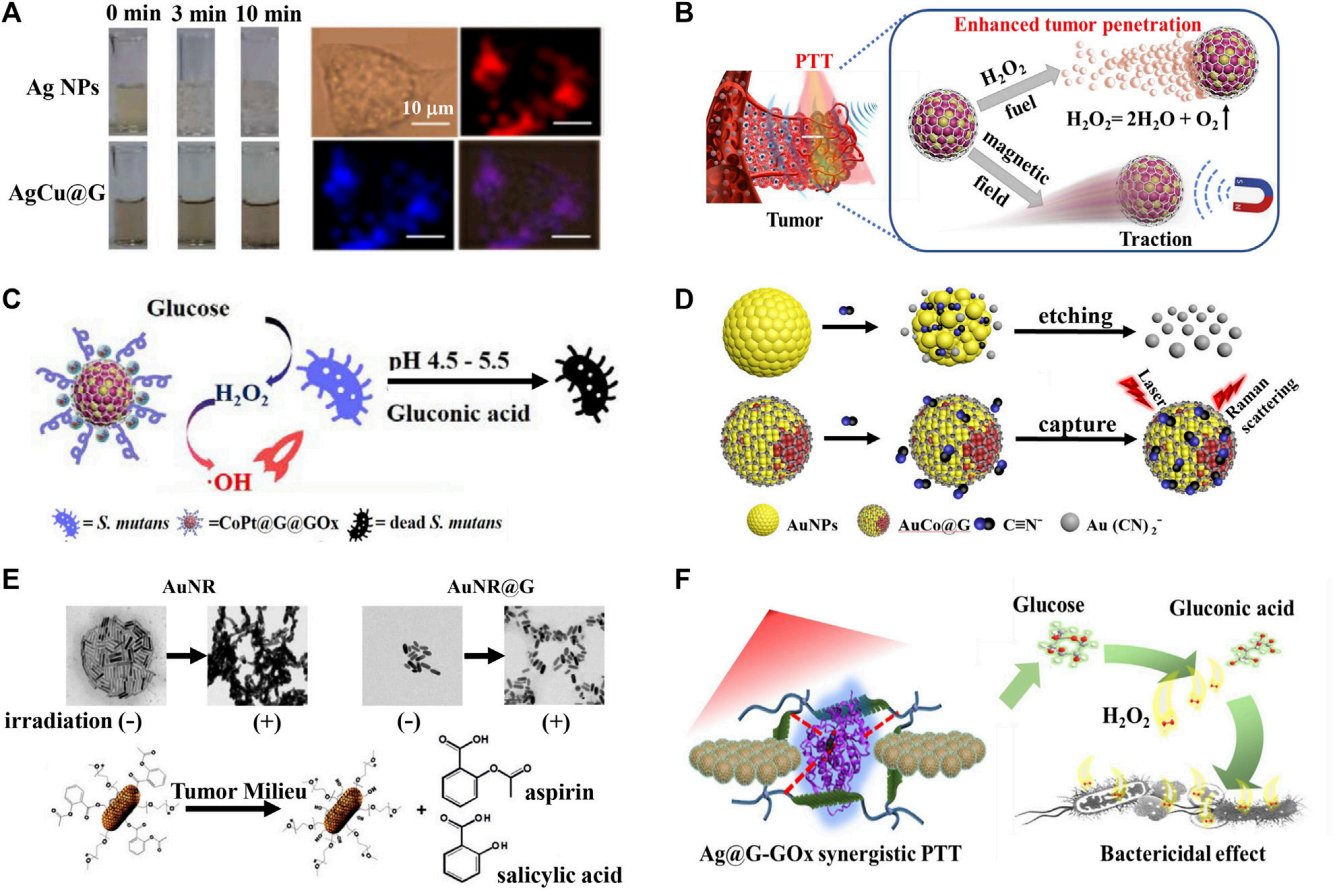
Metal graphitic nanocapsules-based theranostics in non-intrinsic physiological harsh conditions. **(A)** Oxidation resistance of AgCu@G and AgCu@G for multimodal cellular Raman imaging (Song et al., 2014). **(B)** Illustration of the CoPt@G propelled navigator to enhance penetration and PTT action against tumor cells (Zhang et al., 2021a). **(C)** Illustration of the CoPt@G@GOx with magneto-actuated cascade catalytic activity for the therapy of *Streptococcus mutans* biofilm (Dong et al., 2022). **(D)** AuCo@G with corrosion resistance for direct capture and SERS analysis of the CN^−^ (Zhang et al., 2019). **(E)** AuNR@G-P-aspirin for enhanced NIR-mediated PTT of solid tumor and simultaneously inhibit PTT-induced inflammatory response (Dong et al., 2018). **(F)** Thermostable Ag@G-GOx synergistic PTT platform for bacterial elimination (Liu et al., 2022).

Oxidation conditions are able to affect the stability and property of metal NPs, especially the plasmonic-active Ag NPs that are prone to oxidation in air (Han et al., 2011). With the goal of protecting Ag NPs from oxidation, Song et al. (2014) prepared a highly surface enhanced Raman scattering (SERS)-active AgCu graphitic nanocapsules (AgCu@G). Using the intrinsic characteristic Raman bands from the graphitic shell of AgCu@G as the stable Raman label, high-resolution multimodal cellular Raman imaging was achieved (Figure 1A). Latterly, Li et al. (2021) reported a novel AuAg graphitic nanocapsules (AuAg@G) with superior anti-oxidation property and realized SERS quantitative analysis in homogeneous system and multimodal Raman imaging of MCF-7 cells.

Endogenous/exogenous H^+^ and H_2_O_2_ are other kinds corrosive substances that influence the stability and function of metal NPs (Mabilleau et al., 2006). Zhang et al. (2021a) constructed the catalase-like CoPt graphitic nanocapsules (CoPt@G) with superior corrosion resistance to H_2_O_2_, and it catalyzed H_2_O_2_ to produce O_2_, offering a driving force for enhanced tumor penetration. Coupled with the magnetic and photothermal properties of CoPt@G, enhanced penetration and efficient photothermal therapy (PTT) of solid tumors have been achieved with the assistance of H_2_O_2_ and an external magnetic field (Figure 1B). Based on the superior corrosion resistance to H_2_O_2_ of CoPt@G, Dong et al. (2022) further proposed a glucose oxidase loaded CoPt@G (CoPt@G@GOx) platform with cascade reaction activity for *Streptococcus mutans* biofilms treatment. GOx firstly oxidized glucose to generate H_2_O_2_ and gluconic acid in the presence of endogenous glucose. The lower pH of the local microenvironment caused by gluconic acid could enhance the peroxidase-like activity of CoPt@G, and catalyze H_2_O_2_ to produce a large amount of highly toxic •OH, thereby achieving efficient inhibition of bacteria (Figure 1C). Recently, Keoingthong et al. (2021) fabricated a novel peroxidase active Ru graphitic nanocapsules (Ru@G) with superior corrosion resistance to H_2_O_2_ for sensitive colorimetric detection of glutathione (GSH) at near-physiological pH. In the presence of H_2_O_2_, Ru@G could catalyze colorimetric probe 3,3′,5,5′-tetramethylbenzydine (TMB) into blue-colored products, which was inhibited in the presence of GSH, building a simple and sensitive method for the colorimetric detection of GSH. In addition to H^+^ and H_2_O_2_, CN^−^ is also a common corrosive substance that could influence the stability and function of metal NPs-based bioanalytical platform (Wang et al., 2009). Zhang et al. (2019) developed a versatile AuCo graphitic nanocapsules (AuCo@G) with superior SERS activity, magnetic properties and corrosion resistance to CN^−^, and direct SERS analysis of the biomarker of CN^−^ in *Pseudomonas aeruginosa* was achieved (Figure 1D). The superstable AuCo@G proposed a robust platform for detecting *Pseudomonas aeruginosa* infection.

High temperature produced in the PTT process tends to affect the stability of photothermal reagents, meaning the development of stable photothermal reagents is of great significance (Gao et al., 2015). Metal graphitic nanocapsules present excellent thermostability and the graphitic layer has superior spectral absorption properties, thus showing extensive prospects in PTT applications. Dong et al. (2018) found that gold nanorod graphitic nanocapsules (AuNR@G) had superior near-infrared (NIR) light absorption property and had a better thermostability than AuNR. They further integrated AuNR@G with anti-inflammatory prodrugs (AuNR@G-P-aspirin) to realize enhanced NIR-mediated PTT of solid tumor and simultaneously inhibit PTT-induced inflammatory response (Figure 1E). Based on the superior thermostability and photothermal property of AuNR@G, Xu et al. (2019) reported a AuNR@G-doped hydrogel system for highly efficient photothermal antibacterial therapy for both Gram-negative *Escherichia coli* and Gram-positive *Staphylococcus aureus*. Recently, Liu et al. (2022) reported a GOx and Ag graphitic nanocapsules (Ag@G) co-loaded silk membrane theranostic system, the silk membrane enabled GOx to maintain good antibacterial activity during the photothermal antibacterial therapy process, the inert graphitic shell enabled Ag@G to keep excellent SERS performance and photothermal performance under high temperature and GOx-catalyzed production of large amounts of H_2_O_2_, SERS identification of bacteria in the bacteria-infected wound model and efficient synergistic treatment of bacteria were eventually achieved (Figure 1F).

### Theranostics in Harsh Stomach Conditions

Gastric environment is a harsh condition with extremely low pH (0.9-1.5) and abundant pepsin. Among these, H^+^ is a corrosive substance that affects the stability of theranostic reagents, and pepsin in gastric fluid can degrade or bind to theranostic reagents nonspecifically, resulting in instability and inefficiency of theranostics (Huang et al., 2015). Metal graphitic nanocapsules have excellent corrosion resistance to H^+^ and degradation resistance to pepsin, providing a robust platform for the theranostic of gastric diseases.

**FIGURE 2:**
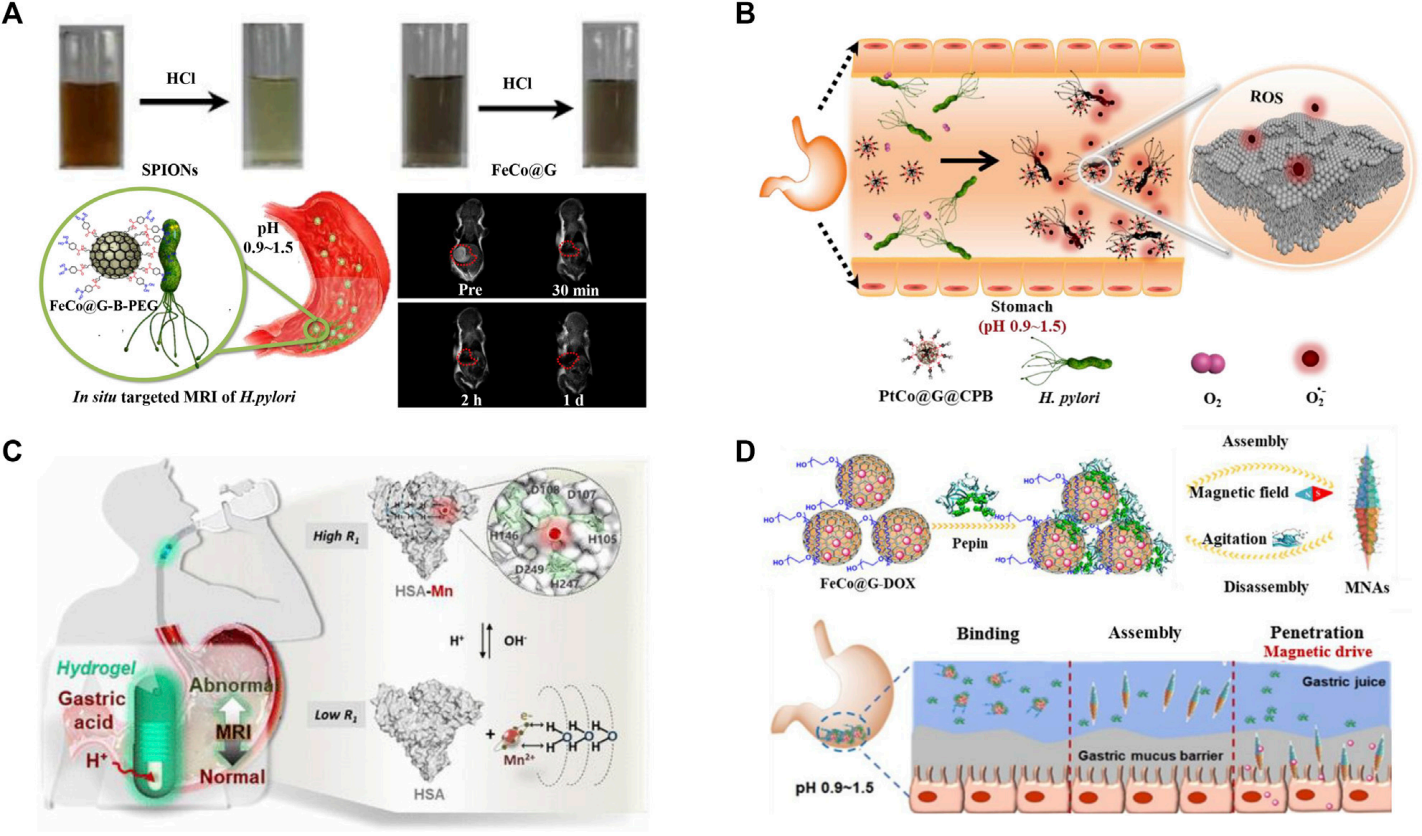
Metal graphitic nanocapsules-based theranostics in harsh stomach conditions. **(A)** Stability of FeCo@G in 1 M HCl and FeCo@G-B-PEG for *in situ* MRI images of *H. pylori* (Li et al., 2017). **(B)** CoPt@G@CPB platform for targeted and selective combating *H. pylori* infection *in vivo* (Zhang et al., 2021b). **(C)** Hydrogel isolated HSA-Mn system for MRI monitoring of gastric pH (Xu et al., 2021). **(D)** Pepsin-assisted assembly of FeCo@G for enhanced mucosal penetration depth and prolonged drug retention (Cai et al., 2021).

*Helicobacter pylori* (*H. pylori*) infection is implicated in the aetiology of many diseases (Sanders and Peura, 2002). Although a series of methods for the detection of *H. pylori* infection have been developed, researchers have never stopped exploring safer and more efficient *in situ* diagnostic methods (Abu Shady et al., 2015; Celik et al., 2021; Jain et al., 2021). Magnetic resonance imaging (MRI), a powerful technique with superior penetration depth, noninvasiveness, high spatial and temporal resolution, shows great promises for the *in situ* detection of *H. pylori* infection (Hsieh et al., 2019). However, the harsh gastric acid environment affects the application of conventional contrast agent such as Gd^3+^ complexes and superparamagnetic iron oxide (Stephen et al., 2011; Li and Meade, 2019). Previous works reported that the FeCo graphitic nanocapsules (FeCo@G) showed superior stability and magnetic properties in solutions with extensive pH range, including the 1 M HCl solution (Chen et al., 2012; Nie et al., 2014). Based on the unique properties of FeCo@G, Li et al. (2017) used the FeCo@G as a robust contrast agent, and further prepared the benzeneboronic acid-PEG (B-PEG, a molecule that could specifically bind to peptidoglycan in bacterial cells) modified FeCo@G system for *in situ* targeted MRI imaging detection of *H. pylori* in mice (Figure 2A). Triple therapy (a proton pump inhibitor and two antibiotics) is used as the standard first-line therapy in the clinical treatment of *H. pylori* infection, but its efficacy is greatly limited by the rapid degradation of antibiotics in gastric acid, the emergence of drug-resistant bacteria and the side effect to the intestinal flora (Wu et al., 2012; Brestoff and Artis, 2013). Nanozyme-based bacterial therapy has been developed rapidly in recent years, and it is expected to provide new options for the treatment of *H. pylori* (Huang et al., 2019; Jiang et al., 2019)*.* Zhang et al. (2021b) developed a bacteria-targeting molecule C_18_-PEG_n_-benzeneboronic acid-functionalized CoPt graphitic nanocapsules (CoPt@G@CPB) platform for targeting and selective combating *H. pylori* infection *in vivo*. The CoPt@G showed superior corrosion resistance in acidic conditions and its oxidase-like activity was activated to catalyze the generation of superoxide radical species for antibacterial applications (Figure 2B). Meanwhile, its oxidase-like activity was suppressed under intestinal neutral conditions, showing minimal side effects. In addition, MRI and Raman imaging was used for monitoring the distribution of CoPt@G@CPB to guide further treatment.

Abundant pepsin in the gastric fluid can also affect the theranostics of gastric diseases because it tends to degrade or bind to the theranostic reagents nonspecifically. Many gastric diseases are highly correlated with abnormal pH, and *in situ* pH monitoring is therefore indispensable for prevention and treatment of gastric diseases (Ma et al., 2021a). To realize accurate and interference-free MRI detection of gastric pH, Xu et al. (2021) developed an orally administrated hydrogel capsule isolated human serum albumin−manganese (HSA-Mn) complex system, which could shield the interference of the pepsin in gastric fluid without severely hindering the penetration of H^+^, for sensitive MRI monitoring of gastric pH *in vivo* (Figure 2C). Magnetic nanomaterials show huge potential for enhanced targeted drug delivery, the FeCo@G with superior corrosion resistance to gastric fluid is therefore expected to be a robust tool for targeted drug delivery in the stomach (Yang et al., 2014; Xu et al., 2018). Cai et al. (2021) surprisingly discovered FeCo@G could not only avoid the interference, but also use the pepsin as a “bridge” to realize the self-assembly of FeCo@G under an external magnetic field, and enhanced mucosal penetration depth and prolonged drug retention time were finally achieved *in vivo* (Figure 2D). This magnetic field-mediated *in situ* self-assembly platform without the interference of extremely acidic and pepsin-rich stomach conditions provided new ideas for the delivery of oral drugs and site-selective treatment of gastric diseases.

## Discussion and Perspectives

Versatile metal graphitic nanocapsules have been widely used for theranostics in harsh conditions due to their excellent stability, good biocompatibility and unique physicochemical properties. In this mini-review, we have summarized the recent advances in metal graphitic nanocapsules for theranostics in different harsh conditions. Firstly, plasmonic metal graphitic nanocapsues that can resist corrosion and high temperature damage have been constructed for reliable SERS bioanalysis, efficient photothermal anticancer and photothermal antibacterial applications in harsh conditions. Secondly, metal graphitic nanocapsules with robust MRI contrast ability and nanozyme activity under strong acid conditions have shown superior performance in the theranostics of gastric diseases. Finally, magnetic and magnetocatalytic propelled metal graphitic nanocapsules that can be stabilized in harsh conditions have been developed as delivery platforms for enhanced gastric mucus penetration and tumor penetration.

Despite great progress has been made in metal graphitic nanocapsules for theranostics in harsh conditions, some critical issues are still needed to be resolved. Firstly, novel multifunctional metal graphitic nanocapsules should be developed to broaden their scope of disease theranostics applications in harsh conditions. Secondly, long-term toxicity of metal graphitic nanocapsules *in vivo* should be systematically explored for promoting expected clinical applications. Finally, the integration of metal graphitic nanocapsules with some advanced technology like Raman endoscope should be considered to acquire more accurate and abundant information for the theranostics of grastric diseases. We expect the superstable metal graphitic nanocapsules will offer robust nanoplatforms for future clinical theranostics without the interference of harsh conditions.
